# Capacity to give informed consent in patients with severe mental disorder in different treatment settings

**DOI:** 10.3389/fpsyt.2025.1709553

**Published:** 2026-01-02

**Authors:** Mounira Jabat, Mona Redlich Bossy, Nikolaus Bausch Becker, Hannah C. Keppeler, Lena Machetanz, Johannes Kirchebner, Stefan Vetter, Erich Seifritz, Stephan T. Egger

**Affiliations:** 1 Faculty of Medicine, University of Zurich, Zürich, Switzerland; 2 Department of Adult Psychiatry, Psychiatric University Hospital Zurich, Zürich, Switzerland; 3 Department of Forensic Psychiatry, Psychiatric University Hospital Zurich, Zürich, Switzerland

**Keywords:** capacity, informed consent, decision making, forensic psychiatry, Hopkins Competency Assessment Test (HCAT), severe mental disorder

## Abstract

**Introduction:**

Individuals with mental disorders, especially those in coercive or forensic settings, face unique vulnerabilities that complicate the process of obtaining informed consent. These challenges stem from both intrinsic factors (such as cognitive impairment due to psychiatric illness) and extrinsic factors (such as institutionalization or legal detention), resulting in what is often referred to as ”dual vulnerability”. This study implemented the German version of the Hopkins Competency Assessment Test (HCAT) and examined differences in informed consent capacity among individuals with severe mental disorders in different treatment setting, aiming to explore how institutional context affects patients’ comprehension and decision-making.

**Methods:**

This study was conducted at the Psychiatric University Hospital Zurich between 2022 and 2023. Using the Hopkins Competency Assessment Test (HCAT), we assessed decision-making capacity in patients with severe mental illnesses, including schizophrenia spectrum and affective disorders, with and without comorbid substance use. Three groups were compared: forensic psychiatric inpatients, non-forensic psychiatric inpatients, and healthy controls.

**Results:**

The study involved 142 participants, mostly male, with a mean age of 37.5 years. Among clinical participants, schizophrenia spectrum disorders predominated, followed by affective disorders, and over a third had comorbid substance use disorders. Forensic psychiatric patients required more time and simpler language to complete the HCAT. They made more errors, showed increased reading effort, and achieved significantly lower comprehension scores compared to the other groups. These differences were not only explained by diagnosis, demographics, or clinical severity.

**Discussion:**

Patients with severe psychiatric disorders, especially in forensic settings, encounter unique challenges in understanding and providing informed consent. These challenges stem from both individual cognitive or clinical impairments and the institutional context, such as coercive environments and complex legal-ethical frameworks. Our findings highlight the need for setting-sensitive communication strategies, legal safeguards, and increased awareness of advance care planning in forensic institutions. Future research should not only assess capacity, but also explore patients’ willingness and opportunity to participate meaningfully in ethical and legal decisions about their care. The study was registered at ClinicalTrials.gov (NCT05939765).

## Introduction

### The issue of informed consent in mental disorder and coercive settings

Both psychiatric patients and detained individuals are considered particularly vulnerable populations. This vulnerability arises from both intrinsic factors, such as severe mental illness (i.e. illness with a high burden of disease, with a chronic or recurrent course leading to impairment, such as Schizophrenia) (1–3), and extrinsic factors, such as institutionalization and imprisonment (4–6). Forensic psychiatric patients are exposed to both, experiencing what is referred to as *dual vulnerability*—the combination of serious mental illness and confinement within highly restrictive (and potentially formally and informally coercive) treatment environments (7, 8). This dual vulnerability poses distinct ethical challenges that go beyond those typically encountered in general psychiatric or clinical research settings (9).

A central concern in this context is whether patients possess the capacity to provide informed written consent. While such capacity is legally presumed in adults (10), it may be compromised by psychiatric disorder or the coercive nature of forensic settings (where higher rates of informal coercion, such as persuasion or inducement exists). Moreover, restrictive legal and ethical frameworks have significantly limited empirical research in forensic psychiatry—often resulting in reliance on case reports and retrospective designs rather than controlled clinical trials (11).

From a legal and ethical point of view, individuals retain the fundamental right to informed consent, particularly in psychiatric treatment and research contexts. This includes the right to refuse interventions (which requires a higher level of assertiveness on the patient’s side). Clinicians must therefore be familiar to the ethical and legal parameters that govern consent processes and ensure that patient autonomy is preserved (12); avoiding informal coercion like in persuasion or inducement. This creates a tension between respecting autonomy and fulfilling the principal of beneficence, especially when cognitive, emotional, or behavioural impairments complicate decision-making. Furthermore, informed consent should not be seen as a one-time act but as an ongoing process that adapts to the patient’s evolving clinical condition (13).

Paradoxically, efforts to protect this vulnerable population by excluding those being kept under detainment from research participation may hinder progress in forensic psychiatric care. Such exclusions limit the development of evidence-based diagnostics and individualized treatment approaches (14, 15). There is a pressing need for more research in this area; particularly across core domains of clinical practice: diagnostic and risk assessment, treatment intervention (both pharmacological and psychological); rehabilitation and integration; and finally, how to prevent coercive measures (16).

From ethical, scientific, and clinical perspectives, it is essential to provide practitioners with brief and validated tools to assess a patient’s capacity to provide informed consent in these settings. One such tool, already evaluated in previous clinical and research contexts, is the Hopkins Competency Assessment Test (HCAT) a standardized screening tool designed to evaluate an individual’s capacity to provide informed consent (17, 18). Further instruments are available, including the MacCat-T, the SICIATRI-R and the EICT (19–21). In contrast to the HCAT these are time- intensive, structured interviews conducted by specialized psychologist or psychiatrist, while the HCAT is conceived as a screening tool capable to be delivered by trained personal. Beyond their complexity, no instrument is capable to replace clinical judgement. Therefore, the HCAT seems to be a more versatile instrument for the day- to- day clinical practice (17).

### Study aims

This study aimed to implement the German version of the Hopkins Competency Assessment Test (HCAT) and to examine differences in informed consent capacity among individuals with severe mental disorders across various treatment settings. Specifically, it compared response patterns between patients in high and low security forensic wards and non-forensic psychiatric inpatient care, as well as healthy controls. High- and low-security wards differ primarily in their approaches to risk management and the preservation of patient autonomy. High- security wards have a standard closed to the penitentiary system including security personal, including a sluice system. Low- security wards are closed but operated solely by therapeutic staff, and are not escape-proof. Forensic psychiatric treatment must take place in the least restrictive environment possible, while simultaneously ensuring adequate security (22).

The goal was to explore if and how institutional context influences patients’ comprehension and decision-making abilities, with the broader aim of improving the assessment of consent capacity in clinical and research settings.

## Materials and methods

### Study design and sample population

Participants were recruited from the Clinic for Forensic Psychiatry and Psychotherapy at the University Hospital of Psychiatry Zurich in 2022 and 2023. Inclusion criteria were a clinical diagnosis of a schizophrenia spectrum disorder, an affective disorder, a substance use disorder as comorbidity, and current inpatient treatment. Forensic patients were admitted either due to court-mandated treatment or because treatment in a less restrictive setting was considered insufficient.

As a comparison group, non-forensic psychiatric patients were recruited from the Centre for Integrative Psychiatry at the same institution. Non- forensic patients were voluntarily in psychiatric treatment in an open ward. These participants were hospitalized and diagnosed with chronic or treatment resistant psychotic disorders, an affective disorder and eventually a comorbid substance use disorder. We consider that substance use disorders ads complexity and severity to an individual case. In addition, a non-clinical control group was recruited via convenience sampling from hospital staff at the University Hospital of Psychiatry Zurich. This group included employees from diverse professional backgrounds such as nursing, social work, administration, and housekeeping, with no declared psychiatric disorder. We collected just basic, non-traceable demographic data; without further mental health inquirement.

All participants were screened for their capacity to provide informed consent as part of the study’s enrolment procedure. This clinical assessment was conducted by the treating psychiatrist or psychotherapist prior to providing detailed study information and requesting written consent. To reduce selection bias, all patients who were willing and able to participate were included, regardless of initial clinical assumptions regarding their decisional capacity. Exclusion criteria comprised acute psychotic symptoms, severe cognitive impairment, insufficient German language proficiency, and refusal to participate.

### Demographic and clinical data

We collected demographic and clinical data for each study participant, including age, sex, marital status, highest level of education attained, and if patients are under legal tutelage. Additionally, primary diagnoses, comorbid conditions, and illness duration were also recorded.

The severity of the mental health impairment at the time of admission was assessed using the *Health of the Nation Outcome Scales* (HoNOS), a clinician-rated tool designed to measure mental health and social functioning, and is frequently used to asses clinical severity and need of care (23). Additionally, on the day of assessment, we systematically extracted and calculated the defined daily dose (DDD) of prescribed psychiatric medication, including antipsychotics, mood stabilizers, benzodiazepines, antidepressants, and other relevant psychotropic drugs. Furthermore, we classified the antipsychotic medication according to their affinity for the D2 Dopamine receptor into Tight Binding (Zuclopenthixol, Haloperidol, Risperidone and Paliperidone) and Partial Antagonist or Loose Binding (Aripiprazol, Amisulpride, Olanzapine, Cariprazine, Clozapine, Quetiapine); and their anticholinergic properties (Olanzapine, Quetiapine and Clozapine) (24, 25).

### Hopkins competency assessment test

The Hopkins Competency Assessment Test (HCAT) is a standardized screening tool designed to evaluate an individual’s capacity to provide informed consent. It comprises two components: (1) a short informational essay outlining the principles of informed consent and durable power of attorney, and (2) a ten-item questionnaire assessing comprehension of the material (17, 18). To accommodate varying levels of reading ability, the essay is available in three versions of increasing simplicity, corresponding to U.S. 12th-grade, 8th-grade, and 6th-grade reading levels. Participants initially received the 12th-grade version. In cases of difficulty or insufficient performance, simplified versions were presented sequentially.

The HCAT was administered by trained clinicians in accordance with the standardized procedure described in the original manual. After participants read the assigned version of the essay, a prerecorded audio version was played to reduce potential bias related to literacy skills. Scoring was based on the participants’ responses to the comprehension questionnaire. As stated in previous studies, a score of over eight out of ten was considered indicative of sufficient decisional capacity (17). If the initial score was seven or lower, the next lower reading-level version was presented, followed by a reassessment. This stepwise procedure continued until the participant either achieved a passing score or completed all three versions without success.

### Statistical analysis

Sample characteristics were analysed using descriptive statistics, including mean, standard deviation, and proportions. Group comparisons for HCAT total and individual item scores were conducted using Analysis of Variance (ANOVA), followed by pairwise t-tests to identify significant differences between specific groups.

Readability was assessed by recording reading time and calculating a reading effort index based on text complexity. The reading complexity index was determined by word length, sentence structure, and overall text length. HCAT performance was measured using the total score, and a performance index was calculated by relating this score to the time required to respond.

Median and interquartile ranges were calculated for reading time, reading effort, response time, and HCAT performance. Group differences were assessed using the Kruskal–Wallis H test, with *post hoc* pairwise comparisons performed using Dunn’s test. Distributional characteristics were further examined by calculating skewness and kurtosis. All variables were visualized graphically (see **Figure 01**) to support interpretation of group differences. We present both parametric and non-parametric results, for better understandability and visualization of the group results.

Logistic regression was used to examine factors associated with HCAT performance and the likelihood of reaching the threshold score over eight on the first attempt. Analyses were adjusted for demographic and clinical characteristics, as well as reading effort and response time.

All statistical analyses and figures were performed using R (version 4.5.1) and R Studio (Version 2025.05.0 + 496).

### Ethics committee approval

The use of collected data, along with anonymized demographic and clinical information, was approved by the ethics committee (BASEC 2021-01123). The study was registered at ClinicalTrials.gov (NCT05939765). The data can be provided upon request.

## Results

### Demographic and clinical data

A total of 142 individuals participated in the study. The sample comprised 78 forensic psychiatric inpatients (22 from high-security and 56 from low- and medium-security wards), 32 non-forensic psychiatric inpatients, and 32 healthy controls. The overall mean age was 37.5 years (SD = 11.8), and the majority of participants were male (n = 117, 82.4%), which is consistent with typical forensic populations. Approximately 47% of the total sample (n = 67) had not completed education beyond the minimal mandatory education of nine years in Switzerland.

Among the clinical subsample (n = 110), the most frequent diagnosis was a schizophrenia spectrum disorder (n = 83, 75.5%), followed by affective disorders (n = 27, 24.5%). Comorbid substance use disorders were present in 39 patients (35.5%). Patients treated in a regular ward had higher doses of prescribed antidepressants; we found no differences in the dose of antipsychotics, mood stabilizers, benzodiazepines, nor in the remaining prescribed medication. The single most prescribed medication was Clozapine, without differences among the groups (X^2^(2,110)=3.548, p=0.17). In the prescribed antipsychotic medication, we found no differences regarding the receptor profile (X^2^(2,110)=4.293, p=0.12), prescription of combination of two or more antipsychotics (X^2^(2,110)=3.340, p=0.19). However, those in the high security wards had a lower prescription rate of Long Acting Injectables (X^2^(2,110)=8.746, p=0.01). Demographic and clinical characteristics by treatment group are shown in **Table 1**.

**Table 1:** Demographic and clinical characteristics of the sample population.

	Total	Controls	Regular	Forensic		Statistic	p
				Low security	High security		
	*n=142*	*n=32*	*n=32*	*n=56*	*n=22*		
	*Mean (SD)*	*Mean (SD)*	*Mean (SD)*	*Mean (SD)*	*Mean (SD)*		
Demographic variables							
Age (years)	37.48 (11.81)	36.44 (12.98)	41.06 (11.38)	36.36 (11.13)	37.14 (12.63)	F(3,138)=1.155	.32
	*n (%)*	*n (%)*	*n (%)*	*n (%)*	*n (%)*		
Sex							
Male	117 (82.4)	24 (75.0)	24 (74.2)	49 (87.5)	20 (90.9)	X^2^(3,142)=4.518	.21
Female	25 (17.6)	8 (25.0)	8 (25.0)	7 (12.5)	2 (9.1)		
Education							
Regular (or lower)	69 (48.6)	13 (37.5)	12 (37.5)	32 (57.1)	13 (59.1)	X^2^(3,142)=5.762	.12
Apprenticeship (or higher)	73 (51.4)	20 (62.5)	20 (62.5)	24 (42,9)	9 (40.9)		
Clinical variables							
Diagnosis							
Psychotic Disorder	83 (58.5)	n.ap.^‡^	20 (62.5)	47 (83.9)	16 (76.2)	X^2^(2,110)=5.159^‡^	.07
Affective Disorder	27 (19.0)	n.ap.^‡^	12 (37.5)	9 (16.1)	6 (23.8)	X^2^(2,110)=5.159^‡^	.07
Comorbid AUD/SUD	39 (35.5)	n.ap.	10 (31.3)	24 (42.9)	5 (22.7)	X^2^(2,110)=3.145^‡^	.21
Legal Tutor	38 (26.8)	n.ap.^‡^	6 (19.4)	23 (42.6)	9 (42.9)	X^2^(2,110)=4.979^‡^	.08
	*Mean (SD)*	*Mean (SD)*	*Mean (SD)*	*Mean (SD)*	*Mean (SD)*		
Duration of Illness (years)	11.39 (7.92)^‡^	n.ap.^‡^	11.44 (6.77)	11.84 (8.96)	10.18 (6.79)	F(2,107)=0.342^‡^	.71
HoNOS	23.45 (7.32)^‡^	n.ap.^‡^	26.66 (5.48)	21.36 (7.06)^a^**	24.14 (8.70)	F(2,107)=5.943^‡^	.004
Medication (in DDD)							
Antipsychotics	1.78 (1.27)^‡^	n.ap.^‡^	1.72 (1.52)	1.90 (1.13)	1.56 (1.22)	F(2,107)=0.616^‡^	.54
Mood Stabilizers	0.24 (0.72)^‡^	n.ap.^‡^	0.28 (0.76)	0.21 (0.78)	0.25 (0.47)	F(2,107)=0.096^‡^	.91
Antidepressants	0.57 (1.27)^‡^	n.ap.^‡^	1.28 (1.71)^b^	0.22 (0.81)^b^ ***	0.45 (1.10)^b^ *	F(2,107)=8.262^‡^	<.001
Benzodiazepines	0.19 (0.44)^‡^	n.ap.^‡^	0.27 (0.43)	0.14 (0.42)	0.23 (0.50)	F(2,107)=1.017^‡^	.37
Other	0.78 (5.47)^‡^	n.ap.^‡^	0.40 (1.69)	0.95 (7.12)	0.91 (4.26)	F(2,107)=0.109^‡^	.88

a low security < high security = regular.

b low security = high security < regular.

*p <.05; ** *p* <.01; ***p <.001.

^‡^Healthy Controls were excluded from analysis.

DDD, Daily Defined Dose.

### HCAT

All 142 participants completed the HCAT, resulting in a total of 206 assessments (**Table 2**). Initially, all participants received the 12th-grade version. Of these, 57 individuals (67.9%) reached a threshold score of over eight on the first attempt. Forty-two participants (29.6%) required the 8th-grade version, and 22 (15.5%) ultimately completed the 6th-grade version. Fourteen participants (16.7%) passed on the second attempt, and 4 (4.7%) passed on the third. Sixteen participants (10.7%) failed to reach the threshold even after all three versions. All healthy controls (n = 32) passed the HCAT on the first attempt. Among forensic patients in high-security wards, 6 (27.3%) failed to meet the cutoff, compared to 8 (14.3%) in low- and medium-security wards and 2 (6.2%) in non-forensic psychiatric wards (**Table 3**).

**Table 2:** Results of the HCAT essay and questionnaire according to the treatment setting.

	Total	Controls	Regular	Forensic		Statistic	p
				Low security	High security		
	*n (%)*	*N=32*	*_3_N=32*	*_3_n=56*	*_3_n=22*		
Total Questionnaires	206	32	42	90	42	
HCAT 12^th^Grade	142 (68.9)	32 (100.0)	32 (100)	56 (100)	22 (100)	X^2^(6,142)=22.81	<.001
HCAT 8^th^Grade	42 (29.6)	0 (0.0)	7 (21.9)	22 (39.3)	13 (59.1)		
HCAT 6^th^Grade	22 (15.5)	0 (0.0)	3 (9.4)	12 (21.4)	7 (31.9)		
Failed	16 (10.7)	0 (0.0)	2 (6.3)	9 (16.1)	5 (22.7)		
	*Mean (SD)*	*Mean (SD)*	*Mean (SD)*	*Mean (SD)*	*Mean (SD)*		
HCAT Total	7.12 (3.16)	10 (0.00)	8.14 (2.33)	6.64 (3.05)	4.93 (3.34)	F(3,138)=18.96	<.001
	*Mean (SD)*	*Mean (SD)*	*Mean (SD)*	*Mean (SD)*	*Mean (SD)*		
Time to Read (minutes)	1.90 (0.95)	1.25 (0.54)	6.24 (7.61)	5.78 (5.89)^a,*^	7.64 (7.64)^a,**^	F(3,138)=4.71	.004
	*Median (IQR)*	*Median (IQR)*	*Median (IQR)*	*Median (IQR)*	*Median (IQR)*		
	2.00 (1.00)	1.00 (0.13)	2.00 (0.25)	2.00 (0.25)	2.00 (0.75)	H(3,142)=36.23	<.001
	*Mean (SD)*	*Mean (SD)*	*Mean (SD)*	*Mean (SD)*	*Mean (SD)*		
Reading Effort	3.11 (1.56)	2.05 (0.88)	13.40 (18.25)	12.78 (14.59)	17.15 918.32)^b,**^	F(3,138)=4.28	.006
	*Median (IQR)*	*Median (IQR)*	*Median (IQR)*	*Median (IQR)*	*Median (IQR)*		
	3.27 (1.64)	1.64 (0.21)	3.24 (0.41)	3.24 (0.82)	3.24 (1.23)	H(3,142)=35.50	<.001
	*Mean (SD)*	*Mean (SD)*	*Mean (SD)*	*Mean (SD)*	*Mean (SD)*		
Time to Answer (minutes)	5.42 (3.19)	3.16 (0.63)	9.32 (6.70)	13.92 (10.77)^c,***^	17.83 (12.28)^c***,d,**^	F(3,138)=11.89	<.001
	*Median (IQR)*	*Median (IQR)*	*Median (IQR)*	*Median (IQR)*	*Median (IQR)*		
	4.00 (4.00)	3.00 (0.70)	3.75 (2.00)	6.00 (5.25)	5.00 (3.00)	H(3,142)=33.46	<.001
	*Mean (SD)*	*Mean (SD)*	*Mean (SD)*	*Mean (SD)*	*Mean (SD)*		
Performance	2.47 (1.21)	3.31 (0.77)	2.42 (1.24)^e^*	1.37 (1.05)^e***^	1.02 (0.68)^e^***	F(3,138)=28.5	<.001
	*Median (IQR)*	*Median (IQR)*	*Median (IQR)*	*Median (IQR)*	*Median (IQR)*		
	2.00 (1.96)	3.33 (0.66)	2.92 (1.27)	1.13 (1.25)	0.73 (1.22)	H(3,142)=62.19	<.001

a low security < high security = control.

b high security < control.

c low security < high security = control.

d high security < regular.

e low security = high security < regular < control.

*p <.05; ** *p* <.01; ***p <.001.

**Table 3:** Results of the single HCAT questionnaire items according to the treatment setting.

Item	Total	Controls	Regular	Forensic		Statistic	p
				Low security	High security		
	n=206	n=32	n=42	n=90	n=42		
01 Procedure	0.69	1.00	0.74	0.62^d***^	0.57^a***^	F(3,202) = 7.10	<.001
02 Shared Decision	0.75	1.00	0.88	0.74^c***,d*^	0.45^a***,b***,c***^	F(3,202) = 13.58	<.001
03 Alternative	0.75	1.00	0.83	0.76^d*^	0.45^a***,b***,c***^	F(3,202) = 12.32	<.001
04 Benefit	0.70	1.00	0.81	0.66^d*^	0.48^a***,b***^	F(3,202) = 10.23	<.001
05 Risk	0.83	1.00	0.95	0.78^d*^	0.69^a**,b**^	F(3,202) = 6.67	<.001
06 Decree	0.80	1.00	0.95	0.77^d*,e*^	0.57^a***,b***,c**^	F(3,202) = 10.83	<.001
07 Representative	0.75	1.00	0.93	0.66^d***,e**^	0.60^a***,b**^	F(3,202) = 10.43	<.001
08 Stipulation	0.62	1.00	0.71^f*^	0.56^d***^	0.38^a***,b**^	F(3,202) = 12.85	<.001
09 Deterioration	0.57	1.00	0.71^f*^	0.48^c***,d*^	0.32 ^a***,b***^	F(3,202) = 17.51	<.001
10 Express	0.70	1.00	0.74 ^f*^	0.70^d**^	0.43^a***,b**,c**^	F(3,202) = 10.89	<.001

a high security < controls.

b high security < regular.

c high security < low security.

d low security < controls.

e low security < regular.

f regular < control.

*p <.05; ** *p* <.01; ***p <.001.

Across all versions and participants, the mean reading time was 5.55 minutes (SD = 6.50), and the mean time to complete the questionnaire was 12.11 minutes (SD = 10.58). Participants who passed the first attempt required significantly less time: 1.90 minutes (SD = 0.95) to read and 5.42 minutes (SD = 3.19) to complete the HCAT. In contrast, those who passed on the second attempt required 5.00 minutes (SD = 1.69) to read and 14.55 minutes (SD = 7.45) to respond. Participants who completed all three versions needed on average 11.32 minutes (SD = 8.84) to read and 20.68 minutes (SD = 12.77) to respond. Even during the first attempt, participants who eventually needed multiple attempts spent more time reading (2.14 minutes, SD = 0.68) and answering (5.86 minutes, SD = 3.55) compared to those who passed immediately.

Reading and response times also varied by treatment setting. Participants in high-security forensic wards spent an average of 1.82 minutes (SD = 0.59) reading the HCAT and 12.15 minutes (SD = 7.63) answering it. In low-/medium-security forensic wards, reading time was 2.16 minutes (SD = 0.78) and answering took 11.12 minutes (SD = 7.93). Non-forensic psychiatric patients spent 1.91 minutes (SD = 0.69) reading and 6.15 minutes (SD = 3.36) answering (**Tables 2** and **3**). Group differences in response time did not reach statistical significance (p = .19). Beyond treatment setting, we found no further significant correlates to effort or performance on the HCAT.

**Figure 1:**
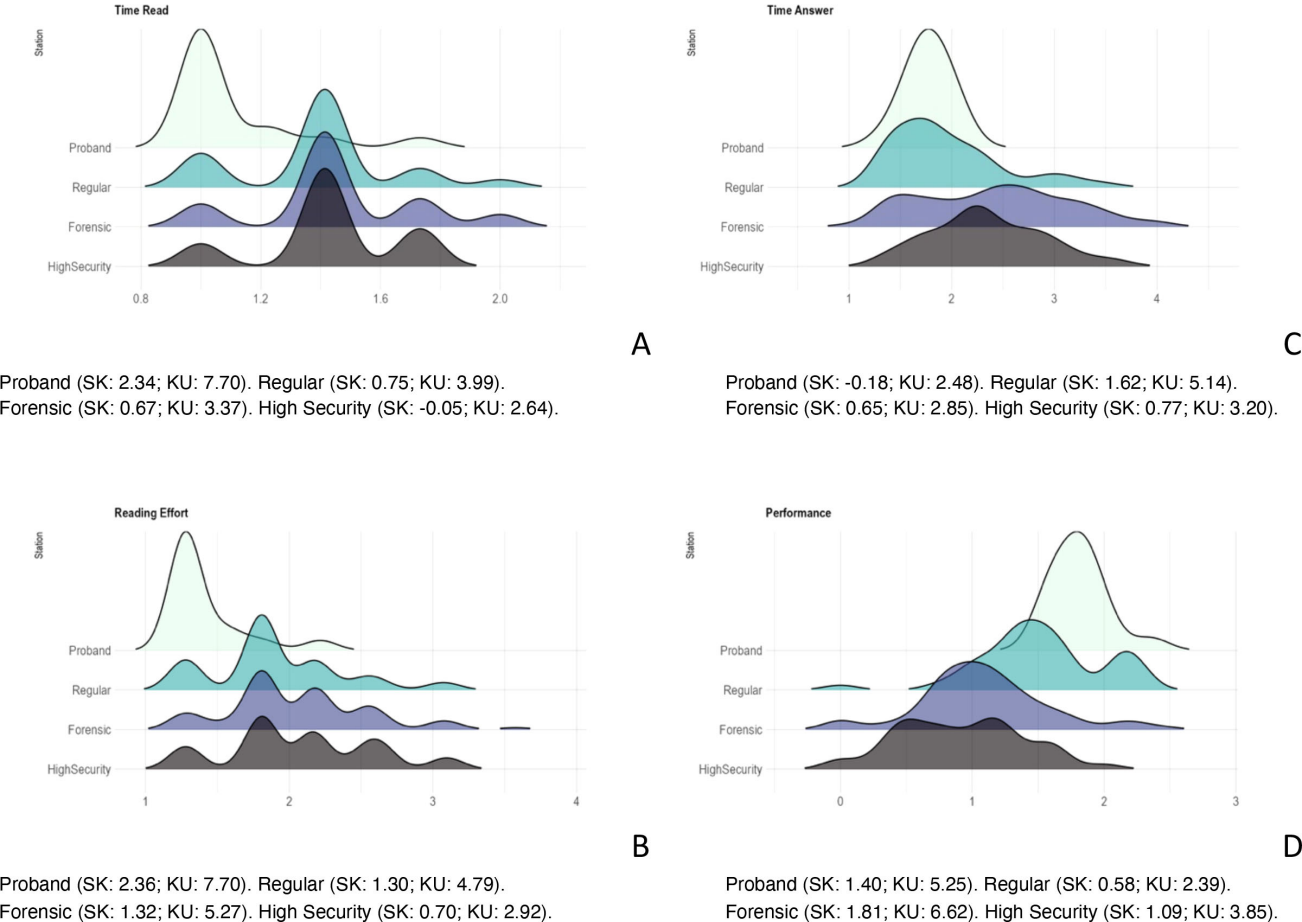
Distribution, skewness (SK) and kurtosis (KU) of the time to read, reading effort, time to answer, and performance indexes according to the treatment setting.

Several differences in the distribution of performance-related variables were observed across groups (see **Figure 1**). The time-to-read variable in the control group showed a leptokurtic, right-skewed distribution, while the patient groups exhibited multimodal patterns, with peaks deviating by up to two standard deviations. Reading effort mirrored this pattern: a right-skewed distribution in controls contrasted with more dispersed, multimodal distributions in clinical groups. Response time was normally distributed among controls but platykurtic and right skewed among patients. Regarding the performance index, controls again showed a leptokurtic, right-tailed distribution, whereas patient groups presented a flatter (platykurtic) distribution.

## Discussion

The present study found that, according to the German version of the HCAT, a smaller proportion of patients in forensic psychiatric care demonstrated adequate decision-making capacity compared to patients from general psychiatry. Forensic patients required more attempts and simpler language versions to reach the comprehension threshold, exerted greater reading effort, and took more time to complete the instrument. Despite these additional efforts, their overall HCAT scores remained lower than those of general psychiatric patients. These findings suggest that, within this sample, forensic psychiatric patients were less likely to meet the cognitive and comprehension requirements associated with informed consent.

However, the reasons underlying this observed difference cannot be directly inferred from the present data. Several potential explanations—spanning illness severity, diagnostic composition, cognitive functioning, and contextual influences—should be considered.

First, differences in clinical severity or functional impairment might partially account for the results. While both patient groups shared comparable psychiatric diagnoses, forensic patients may exhibit higher degrees of chronicity, poorer psychosocial functioning, or more persistent cognitive deficits. These factors, commonly captured in instruments such as the Health of the Nation Outcome Scales (HoNOS), could contribute to reduced comprehension and decision-making ability. Previous studies have shown that impairments in executive functioning and cognitive flexibility are particularly common in chronic psychotic disorders, potentially interfering with information processing during complex consent tasks (26, 27).

Second, diagnostic differences may play a role. The general psychiatric group included more patients with affective disorders, particularly depression, whereas the forensic group contained a higher proportion of patients with psychotic or personality disorders. Depressive symptoms may influence motivation and self-evaluation, but do not typically impair comprehension to the same extent as psychotic disorganization or cognitive deficits associated with schizophrenia-spectrum disorders. Forensic samples also tend to have higher rates of comorbid substance use and antisocial traits, which can further compromise cognitive control and attentional capacity (28–30). Therefore, the diagnostic composition may contribute to—but cannot fully explain—the observed differences in HCAT performance.

Third, contextual and institutional factors specific to forensic psychiatry likely exert a substantial influence. Forensic settings differ from general psychiatric care not only in terms of legal status but also in their therapeutic atmosphere, degree of coercion, and environmental restrictions. Patients are frequently subjected to long-term involuntary treatment under high-security conditions, often with limited autonomy in daily routines. This restrictive context may foster learned passivity, distrust toward institutional procedures, and diminished motivation to engage in tasks perceived as evaluative or administrative (31, 32). Furthermore, therapy fatigue—a state of mental and emotional exhaustion from prolonged treatment and repeated assessments—has been identified as a significant issue in forensic psychiatry (33). It may also generalize to psychological or neuropsychological testing, thereby affecting concentration, task engagement, and ultimately test performance. In addition, fatigue and frustration can erode adherence to rules and routines, which are known to be crucial for therapeutic progress and rehabilitation (34).

The current findings should also be interpreted in light of what HCAT scores actually measure. The HCAT primarily assesses reading comprehension and verbal reasoning in the context of consent-related material, rather than the broader construct of decision-making capacity as conceptualized by Appelbaum and Grisso (10). A lower HCAT score, therefore, does not necessarily indicate a true incapacity to consent, but may instead reflect limited literacy, cognitive fatigue, language comprehension difficulties, or reduced motivation. Moreover, the meaning and implications of HCAT results differ substantially between treatment and research contexts. Consent to treatment involves the patient’s autonomy and capacity to participate actively in therapeutic decisions, whereas consent to research pertains to voluntary participation in scientific inquiry with distinct ethical safeguards and informational demands (35). Thus, HCAT results should not be overgeneralized without considering the specific consent context in which they are applied.

Despite similar diagnostic and severity levels between the groups, forensic patients exhibited reduced awareness of treatment alternatives, shared decision-making processes, and personal autonomy. They appeared less familiar with patient rights, advance directives, and the capacity to weigh risks and benefits of interventions. These findings align with earlier research indicating that the forensic environment itself can impose structural barriers to autonomy and participation, beyond the influence of individual psychopathology (36, 37). Willingness to participate in decision-making may increase as patients approach release, but tends to remain lower during earlier, more restrictive treatment phases (38).

Regarding pharmacological influences, polypharmacy was common across both treatment settings, consistent with previous research (39). Although higher doses of antidepressants were observed among non-forensic patients, these differences likely reflect diagnostic variability rather than serving as a primary explanation for group disparities in HCAT scores. Moreover, the statistical models accounted for medication effects and various levels (drug, dose and receptor profile), suggesting that pharmacotherapy alone does not fully explain the observed performance gap. The only difference we found regarding antipsychotic medication was the lower rate of long acting injectables found in the high security wards, what might suggest a still ongoing, and thus not stable medical treatment.

From a methodological view, one of this study’s main strengths lies in its naturalistic design and the inclusion of a broad, unselected clinical sample across multiple levels of treatment restriction—from general psychiatric wards to high-security forensic units. This diversity allowed for an ecologically valid exploration of contextual influences on decision-making capacity. Nevertheless, the study also has limitations. The HCAT, while standardized and reliable, captures only a narrow dimension of cognitive capacity, focusing primarily on reading comprehension rather than reasoning, appreciation, or the ability to express a choice.

Future research should therefore incorporate multidimensional tools to capture the full complexity of decision-making competence (10). Additionally, the healthy control group in this study was likely composed of individuals with above-average literacy and education, which may limit generalizability and exaggerate differences relative to patient samples.

Taken together, the results underscore that lower HCAT performance among forensic patients should not be equated with an intrinsic or generalized incapacity to consent. Rather, it highlights the need to address contextual and communicative barriers that impede comprehension and participation. Therefore, we consider the use of easy to use and time efficient standardized instruments (such as the HCAT) for screening and assessment of legal capacity essential in psychiatric research and clinical practice. Interventions to improve consent competence should include simplified information materials, the use of visual and multimodal aids, extended discussion time, and systematic education about patient rights and decision-making processes. Training clinical staff to recognize and mitigate contextual barriers is equally essential for upholding ethical standards in forensic psychiatry.

In conclusion, patients with severe psychiatric disorders—particularly those in forensic settings—face unique and multifaceted challenges in understanding and providing informed consent. These challenges arise not only from individual cognitive or clinical factors but also from the institutional context in which care occurs. Ethical research and treatment in forensic psychiatry depend on respecting and supporting patient autonomy through clear communication, contextual sensitivity, and structured institutional safeguards (40–42). Importantly, decision-making capacity should be viewed as a dynamic, context-dependent construct rather than a fixed trait—one that can be enhanced through targeted education, therapeutic engagement, and the consistent reinforcement of patients’ rights and agency.

## Data Availability

The raw data supporting the conclusions of this article will be made available by the authors, without undue reservation.
